# Intravitreal Aflibercept Injection and Photodynamic Treatment of a Patient with Unilateral Subretinal Neovascular Membrane Associated with Fundus Flavimaculatus

**DOI:** 10.1155/2015/748420

**Published:** 2015-03-02

**Authors:** Ali Osman Saatci, Ziya Ayhan, Bora Yüksel, Göktuğ Seymenoğlu, Seenu M. Hariprasad

**Affiliations:** ^1^Department of Ophthalmology, Dokuz Eylül University, 35340 Izmir, Turkey; ^2^Bozyaka Education and Research Hospital, 35170 Izmir, Turkey; ^3^Department of Ophthalmology, Celal Bayar University, 45030 Manisa, Turkey; ^4^Department of Ophthalmology and Visual Sciences, University of Chicago, Chicago, IL 60637, USA

## Abstract

We report the good outcome of combined intravitreal aflibercept injection and photodynamic treatment in a case with fundus flavimaculatus (FFM) and unilateral subretinal neovascular membrane (SRNM). A 57-year-old man with FFM and unilateral SRNM who was treated with two consecutive ranibizumab injections with no improvement at another institution was referred to us. He was treated successfully with three aflibercept injections three months apart and a single photodynamic treatment performed a week after the initial aflibercept injection. Six weeks after the last aflibercept injection visual acuity was improved and submacular exudation exhibited dramatic resolution with a moderate degree of residual scarring. SRNM formation is rarely observed in eyes with FFM and a satisfactory outcome can be achieved with a proper treatment.

## 1. Introduction

Fundus flavimaculatus (FFM) is characterised by the fishtail-like or round, white-yellowish flecks confined to the retinal pigment epithelium that are scattered diffusely throughout the posterior pole and extend out to the midperiphery and the related visual symptoms commence between the third and fourth decade of life and progress slowly [1–3].

During the course of the disease progressive macular atrophy is a much more common finding than the rarely diagnosed subretinal neovascular membrane [4–14]. Hereby, we reported a 57-year-old man with FFM and unilateral occult subretinal neovascular membrane (SRNM) who was treated successfully with three consecutive aflibercept injections and a single session of photodynamic therapy.

## 2. Report of a Case

**Figure 1:**
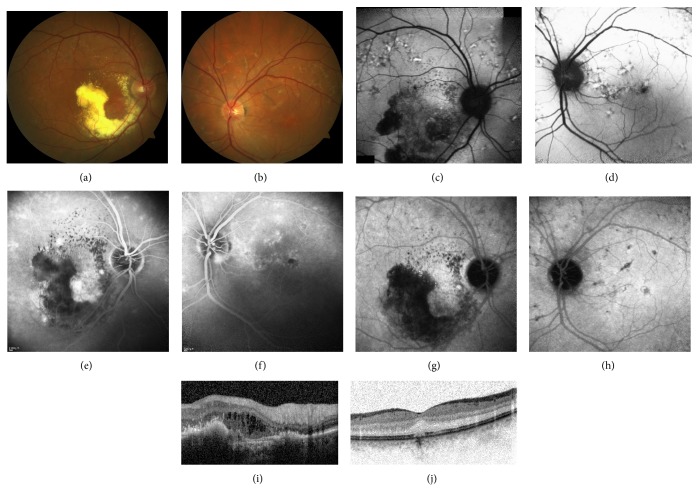
Colour fundus images of OD (a) and OS (b), fundus autofluorescent images of OD (c) and OS (d), venous phase of fluorescein angiography of OD (e) and OS (f), Indocyanine Green angiographic appearance of OD (g) and OS (h), and OCT sections of OD (i) and OS (j) at the time of our initial examination.

A 57-year-old man had a nine-month history of visual decline in the right eye and had received two intravitreal ranibizumab injections for presumed exudative macular degeneration elsewhere. He was referred to us for further evaluation. His family and medical history were unremarkable. On our examination, his best-corrected visual acuity was counting fingers at 2 meters in OD and 20/25 in OS. Slit-lamp examination was normal OU. Fundus examination showed multiple widespread pisciform flecks throughout the posterior pole and midperipheral retina in OU with severe subfoveal exudation and intraretinal fluid in OD (Figures 1(a) and 1(b)). Autofluorescence imaging showed multiple autofluorescent flecks in the posterior pole and perimacular areas (Figures 1(c) and 1(d)). Fluorescein and indocyanine green angiographies exhibited these flecks bilaterally and disclosed an occult type of SRNM with significant leakage in OD (Figures 1(e), 1(f), 1(g), and 1(h)). OCT disclosed severe intraretinal fluid and hyperreflective dots corresponding to hard exudates in OD (Figure 1(i)) while the left eye was unremarkable (Figure 1(j)).

**Figure 2:**
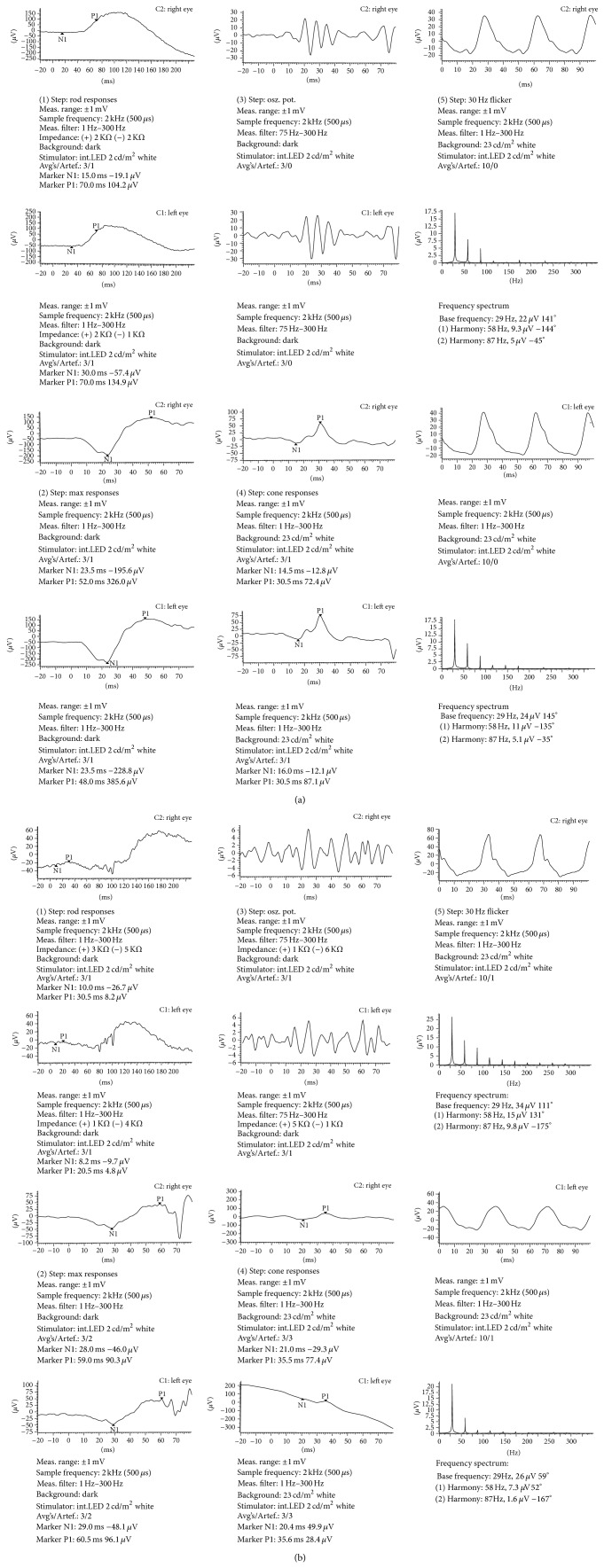
(a) Flash ERG studies of both eyes of a normal control. (b) Flash ERG findings of both eyes of the patient demonstrating the subnormal responses of dark adapted rods, maximum responses, photopic cone responses, and 30 Hz flicker stimulation conditions.

ERG demonstrated subnormal dark adapted responses of rods while photopic cone responses were mildly reduced with prolonged implicit times. Both scotopic 30 Hz flicker and maximum responses were also decreased in amplitude (Figure 2). Our diagnosis was right SRNM associated with fundus flavimaculatus.

**Figure 3:**
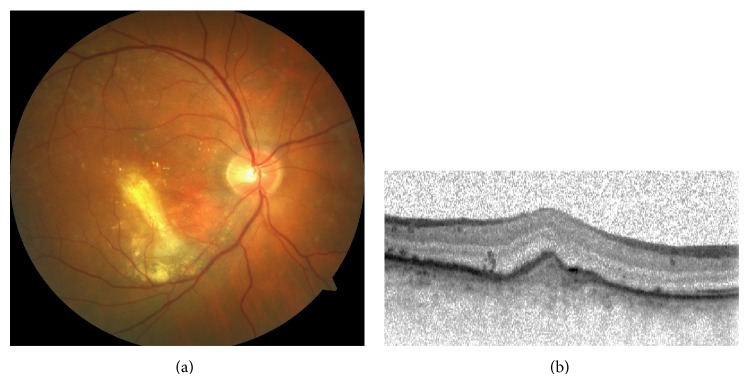
Colour fundus image of right eye (a) and OCT section (b) six weeks after the third aflibercept injection.

Due to unsatisfactory anatomic and visual outcome with two previous ranibizumab injections and the presence of large feeder vessel-like changes noted in the fluorescein and indocyanine green angiographies, we elected proceeding with a combination of photodynamic therapy and aflibercept injection. We preferred aflibercept over ranibizumab as the patient might not be followed up monthly due to his profession. Seven days after the administration of initial 2 mg aflibercept injection standard photodynamic treatment was performed in OD, namely, 6 mg/m^2^ verteporfin injected intravenously over a period of 10 minutes followed by the occlusion of lesion area five minutes later with a 689 nm diode laser for 83 seconds with 50 J/cm^2^ at an intensity of 600 mw/cm^2^. A spot size of 3500 *μ*m was used. Two additional 2 mg intravitreal aflibercept injections were given three months apart. Six weeks after the third injection, his best-corrected visual acuity was 20/200 and subretinal exudation showed dramatic resolution with a residual scar in the OD (Figures 3(a) and 3(b)).

## 3. Discussion

There were anecdotal case reports discussing the place of several treatment modalities and describing the outcome of patients with SRNM in association with FFM. In earlier reports, either observation [4–6, 9] or laser photocoagulation [4, 10] was described as first-line therapeutic approach. Later reports showed some treatment benefit of photodynamic treatment (PDT) or intravitreal ranibizumab injection. Valmaggia et al. [7] applied photodynamic treatment over a predominantly classic subfoveal neovascular membrane associated with FFM and no recurrence was observed during the follow-up of nine months. Souied and colleagues [8] shared their observations on three eyes with FFM and subretinal neovascular membrane treated with PDT. In one eye (with a well-defined membrane) one, in the second eye (with a minimally classic membrane) two, and in the remaining eye (with an occult membrane) three sessions of PDT were performed. In these three cases PDT stopped the leakage and prevented the progression of SRNM. Visual acuity was improved at the end of follow-up period ranging from 15 to 24 months in these eyes. Braun et al. [11] performed two sessions of PDT (together with 4 mg intravitreal triamcinolone acetonide injection at the time of second PDT session) on a 47-year-old patient with classic choroidal neovascularization. After nine months, visual acuity stabilised. However, scarring occurred despite the cessation of leakage.

Tejerina and friends [12] injected ranibizumab intravitreally twice for a subretinal neovascular membrane associated with FFM. Nine months later, visual acuity was improved and no leakage was observed. Quijano et al. [13] diagnosed a type 3 subretinal neovascular membrane in a 78-year-old woman and administered three consecutive intravitreal ranibizumab injections and the visual acuity improved to 20/32 from the initial level of 14/20 six months after the last injection. Koh et al. [14] achieved an excellent visual outcome of 6/6 vision in a 30-year-old Chinese female with a juxtafoveolar subretinal neovascular membrane and FFM with only a single ranibizumab injection.

Intravitreal aflibercept treatment has been proven to be efficacious in exudative age-related macular degeneration [15]. Moreover, in eyes recalcitrant to other anti-VEGF agents, intravitreal aflibercept injection at least provided anatomical benefit [16–18]. Our patient showed a suboptimal response to ranibizumab therapy and we obtained a satisfactory outcome after switching treatment to three consecutive 2 mg aflibercept injections and a single photodynamic treatment despite the presence of heavy subretinal exudation prior to treatment.
